# Microscope‐Free Analyte Detection Based on Fiber‐Optic Gliding Motility Assays

**DOI:** 10.1002/smll.202411836

**Published:** 2025-04-16

**Authors:** Henry Carey‐Morgan, Nabarun Polley, Till Korten, Claudia Pacholski, Stefan Diez

**Affiliations:** ^1^ B CUBE – Center for Molecular Bioengineering TUD University of Technology Dresden 01307 Dresden Germany; ^2^ Institute of Chemistry Physical Chemistry – innoFSPEC University of Potsdam Am Mühlenberg 3 14476 Potsdam Germany; ^3^ Max Planck Institute for Molecular Cell Biology and Genetics 01307 Dresden Germany; ^4^ Cluster of Excellence Physics of Life TUD University of Technology Dresden 01062 Dresden Germany; ^5^ Helmholtz Zentrum Dresden Rossendorf HZDR 01328 Dresden Germany

**Keywords:** antibodies, kinesin, label‐free detection, microtubule, optical fiber

## Abstract

Prolonged hospital waiting times are linked with increased patient mortality and cause additional financial burdens on institutions. Efficient point‐of‐care diagnosis would help alleviate this, but is hampered by a lack of cost‐effective devices capable of rapid, in situ, wide ranging analyte detection. Lab‐on‐fiber technology provides an answer allowing for diagnosis, treatment, and monitoring in situ with real time feedback. Here, a device is demonstrated that harnesses motor‐protein‐driven‐microtubule molecular detection assays to optical fibers. By developing a new method for microscope‐free microtubule gliding speed determination, proof of concept is demonstrated in the detection of Monomeric Streptavidin and Neutravidin, which initiate a decrease in speed in biotinylated microtubules as well as bundling in the latter case. Utilizing antibody functionalizsed microtubules label‐free and microscope‐free detection of the heart attack marker Creatine Kinase‐MB, as well secondary antibodies in nm concentration is demonstrated. This detector has the potential to be used in situ, providing rapid, low‐cost, multiplex analyte screening and detection.

## Graphical abstract

Lab‐on‐fiber technology enables rapid, cost‐effective, and in situ analyte detection, addressing critical gaps in point‐of‐care diagnostics. This study presents a microscope‐free biosensor that utilizes motor‐protein‐driven microtubules on optical fibers to detect biomarkers like Creatine Kinase‐MB. By tracking gliding speed changes, it offers a novel, label‐free detection method with real‐time feedback, enhancing medical diagnostics.

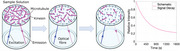

## 1 Introduction

Gliding motility assays, based on the propulsion of microtubules over a substrate by surface‐bound kinesin motors, have been shown capable of cargo pick‐up and analyte detection through biochemical functionalization.^[^ ^1^, ^2^, ^3^, ^4^, ^5^, ^6^, ^7^, ^8^, ^9^, ^10^, ^11^, ^12^, ^13^ ^]^ Conjugating microtubules with biotin or antibodies allows for the loading of analyte‐cargo, which affects microtubule gliding in two ways. First, binding molecules larger than 10 kDa causes a decrease in speed due to the roadblock effect whereby kinesin is obstructed by bound molecules blocking their path; decreases in speed by up to 85% have been observed.^[^ ^6^, ^14^ ^]^ A second behavior cargo‐loaded‐microtubules exhibit is that should the cargo have multiple binding sites, microtubules will form bundles as they are cross‐linked via the analyte in as low as pm concentration.^[^ ^11^ ^]^ Observing these phenomena allows for the detection of analytes present in the sample solution through which the microtubules are gliding. However, so far, these phenomena have only been measured with bulky, expensive optical microscopy setups. This limits the usefulness of the methods, for example in clinical settings where point‐of‐care diagnostics can decrease waiting times by removing the transportation of samples to a central lab as well as by prefiltering patient load.^[^ ^15^, ^16^, ^17^, ^18^, ^19^ ^]^ One field of interest for point of care is the diagnosis of heart disease, which is currently the leading cause of fatalities worldwide, making up 16% of total deaths.^[^ ^20^ ^]^ In fact, 22%–60% of heart attacks are “silent” presenting minor or no obvious symptoms, while on the other hand, only 3% of people who present to primary care with chest pains are actually having a heart attack.^[^ ^21^, ^22^, ^23^ ^]^ Fast, accurate diagnosis and filtering of patients at point‐of‐care is, therefore, crucial.

Here we demonstrate a portable device coupling the analyte detection capabilities of gliding motility assays to optical fibers, a cost‐effective, compact technology already in use for biosensing in combination with other materials and systems.^[^ ^24^, ^25^ ^]^ In particular, we use fluorescently labelled, antibody‐functionalized microtubules and perform gliding motility assays on the polished, flat tip of an optical fiber. We detect the fluorescent signal of the microtubules through the fiber and quantify the decay of this signal over time as the microtubules leave the fiber tip. This decay then informs about the behavior of the microtubules, such as their potential decrease in gliding velocity and/or microtubule–microtubule bundling due to the presence of specific analytes in the sample solution. As we will show, our device is capable of wide‐ranging molecular recognition due to its being underpinned by the use of antibodies. Therefore, it is able to detect any analyte for which there is an antibody, as long as said analyte is present in concentrations within the detection limit and has a large enough molecular weight to impact microtubule gliding.

## 2 Results

**Figure 1:**
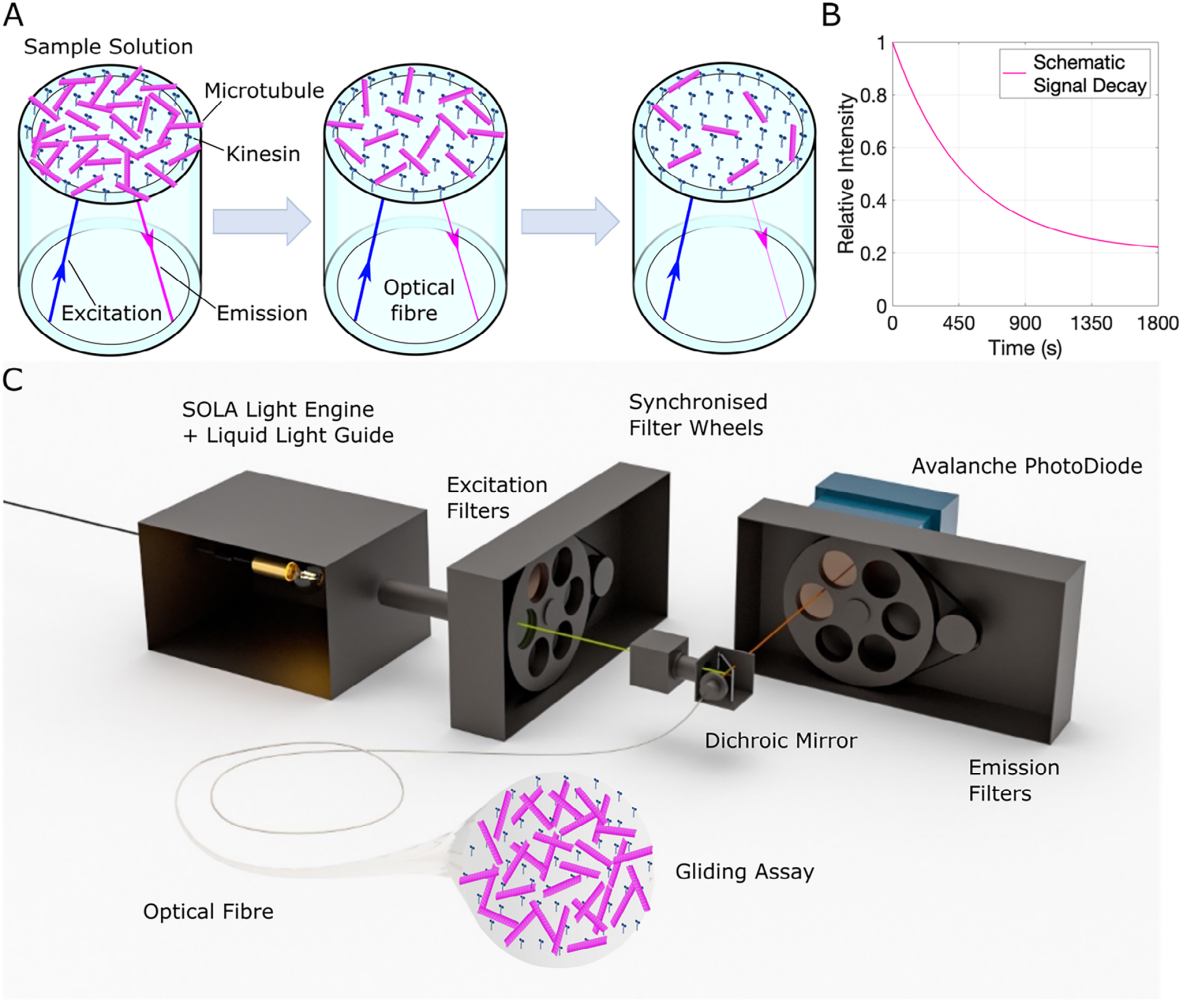
Fibre Optic molecular PRobe at point‐OF‐care (FOOLPROOF). a) Fluorescently labelled microtubules glide on a bed of kinesin‐1 adhered to the flat tip of an optical fiber and are illuminated by excitation light (navy) passing through the fiber via total internal reflection. The gliding assay is mounted onto the fiber by dipping the fiber tip into successive solutions of BRB80‐Casein, Kinesin Solution and Microtubule Solution (**Experimental Section**). The fiber is then dipped into Low‐ATP Motility solution as a wash step to remove microtubules in solution around the fiber, and finally into High‐ATP Motility solution in which the measurement is conducted. Over time the number of microtubules decreases as they are crowd‐surfed off of the edge of the fiber. Their fluorescence emission signal (magenta), part of which travels back down the fiber, also decreases. b) Schematic of expected relative fluorescence signal decay over time as microtubules are propelled off of the tip of the optical fiber. c) Render of the detector. The detector is made up of a SOLA light engine with a shutter, a liquid light guide, a collimator, two filter wheels containing fluorophore excitation and emission filters, a multiband dichroic, a multimode 200 µm diameter optical fiber, and an avalanche photodiode. When a measurement is taken the shutter at the light source opens for 100 ms. White light is then collimated and filtered to the excitation wavelength of the fluorophore labelling the microtubules gliding on the fiber tip. This excitation signal (blue) is reflected by a multiband dichroic through the fiber to the gliding assay where the microtubules fluoresce. A portion of the emission signal (magenta) travels back down the fiber. This light is transmitted through the dichroic and an emission filter to the avalanche photodiode which measures the signal intensity. The shutter closes.

To perform fiber‐optic gliding motility assays, we coated the fiber by dipping 8 mm of the fiber tip length into successive solutions of BRB80‐casein for 5 min, kinesin‐1 solution for 10 min, and microtubule containing motility solution for 10 min (Experimental Section). The fiber tip was then washed in low‐ATP motility solution to remove unbound microtubules and placed into an analyte‐containing motility solution in which the measurement is conducted. Over time and in the presence of ATP the number of microtubules on the core of the fiber tip decreases as they are pushed onto the cladding and off of the edge of the fiber (**Figure** **1**A). Any fluorescence emission signal they produce, therefore, also decreases (Figure 1B). It is this signal that allows us to make a detection as gliding speed is related to the rate of decay of the fluorescence emission signal; fast‐moving microtubules will leave the fiber quickly, and their emission signal will rapidly decrease, whereas a slow‐moving population's emission signal will decrease more slowly.

The gliding assay is illuminated, and the emission signal is detected by the optical‐mechanical setup (Figure 1C). The detector is made up of a SOLA light engine with a shutter, a liquid light guide, a collimator, two filter wheels containing fluorophore excitation and emission filters, a multiband dichroic, the multimode 200 µm diameter optical fiber, and an avalanche photodiode (APD). White light is filtered to the specific wavelengths of the fluorescent dye coating the microtubules, reflected at the dichroic, and taken to the gliding assay via total internal reflection. The microtubules fluoresce, and a portion of the emission signal moves back through the fiber. This is then transmitted by the dichroic, filtered, and captured by an avalanche photodiode, converting the photon number to an electrical signal computed by LabView software. This signal decays with time as the microtubule population on the fiber decreases.

**Figure 2:**
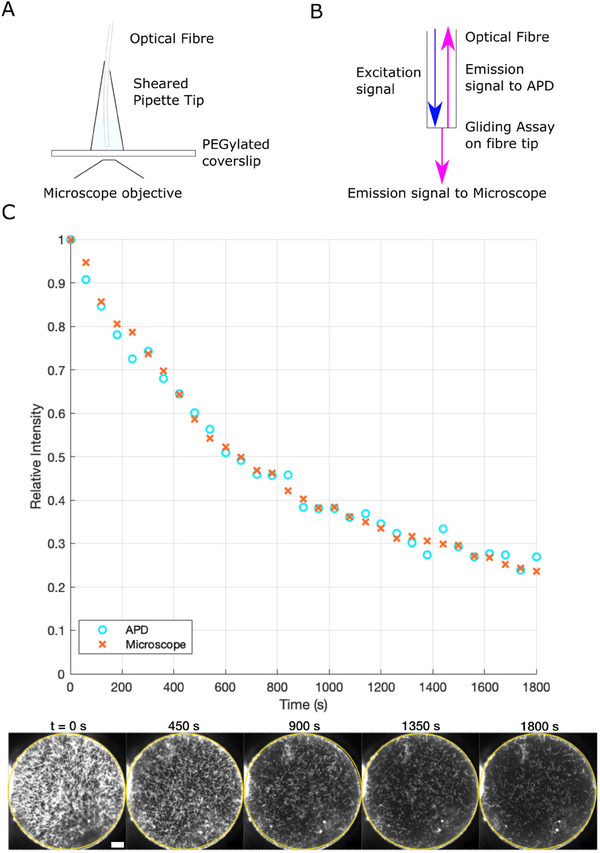
Confirmation of Gliding Assay adherence and Avalanche Photodiode signal by imaging the fiber tip with a microscope. a) Schematic of TipCell setup for imaging gliding assays mounted on fiber. A pipette tip is sheared to just larger than the optical fiber diameter and affixed to a PEGylated coverslip using UV‐activated adhesive (Norland 61). Microtubule Motility solution (1 mm ATP, 20 mm d‐glucose, 20 mm glucose oxidase, 10 mm catalase, 10 mm DTT, and 10 µm Taxol in BRB80) was inserted into the tip cell using a syringe. b) Schematic of fiber tip in TipCell with excitation light (navy) arriving from the fiber and exciting the fluorophore‐labelled microtubules. A portion of the emission signal (magenta) travels back down the fiber to the avalanche photodiode and a portion through the microscope objective to the EMCDD Camera. c) Relative intensity signal of Alexa647 labelled microtubules on a 200 µm diameter fiber measured with the avalanche photodiode and LabView software and corroborated with FIJI software operating on videos taken of the fiber tip, measuring intensity within the yellow circle. Every point shown in the avalanche photodiode signal is the average of 198 data points taken over the course of 58 s. When the shutter opens for 100 ms the APD records the signal strength every 10 ms giving nine points as the shutter opening is not instantaneous. The shutter opens and closes 22 times in 58 s. Images show fiber at Time = 0, 450, 900, 1350 and 1800 s. Scale bar 20 µm.

To verify that the decreasing fluorescence emission signal measured by the avalanche photodiode corresponded to the decreasing number of microtubules, we imaged a gliding assay‐on‐fiber with Alexa647‐labelled microtubules, positioning it under a microscope objective and securing it in place with a “TipCell” (**Figure** **2**A). The assay is still only illuminated by light from the prototype detector's light source traveling down the fiber, not from the microscope, allowing us to see what is occurring on the fiber tip and to take measurements of the decaying fluorescence signal simultaneously (Figure 2B; Movie S1, Supporting Information). The relative intensity, measured in the videos of the fiber tip using FIJI in the region of interest encompassing the entire core of the fiber, decays at the same rate as the signal measured by the avalanche photodiode. Every point shown in the avalanche photodiode signal is the average of 198 data points taken over the course of 58 s. When the shutter opens for 100 ms the APD records the signal strength every 10 ms, giving nine points as the shutter opening is not instantaneous. The shutter opens and closes 22 times in 58 s (Figure 2C). This corroboration of the signal decay confirms that we measure the decaying microtubule population with the avalanche photodiode signal.

**Figure 3:**
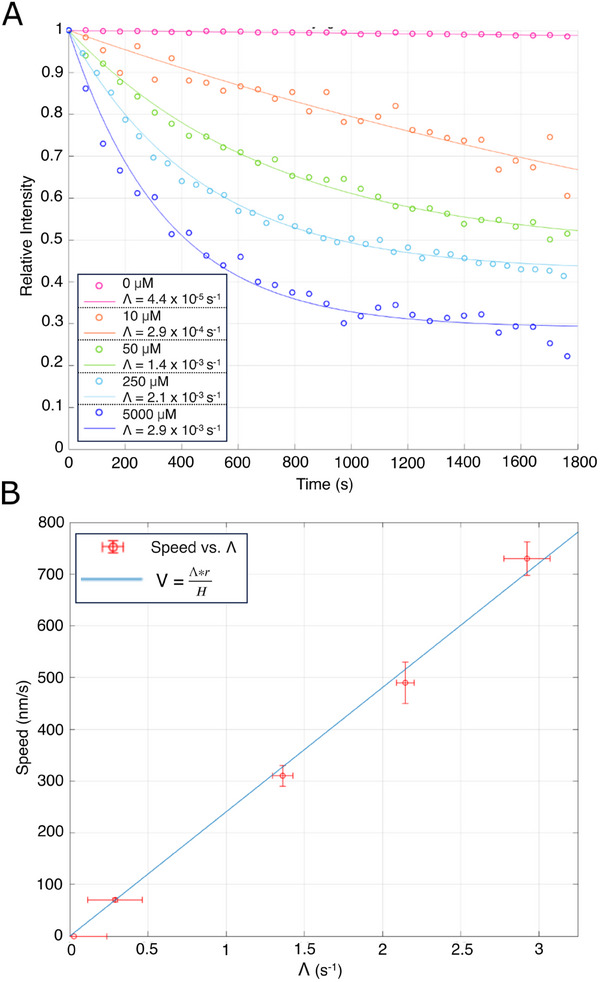
Decay curves of Alexa647 labelled microtubules at a range of different ATP concentrations with decay constants plotted against speed. a) As expected, microtubules moving at slower speeds leave the fiber more slowly so their population and emission signal decays at a lower rate. The decay curves are fitted with a mathematical model composed of an exponential decay of the microtubule population on the tip of the fiber moderated by the inverse population of microtubules that have left and re‐joined the fiber core – Equation (3). b) The value of the decay constant Λ can then be related to gliding speed as in Equation (4). By plotting our Λ values against microtubule gliding speed *V* measured independently using FIESTA tracking software on videos of the fiber tips, we confirm the linear Λ – v relationship.

To demonstrate the change in the rate of signal decay with altered microtubule speed, we performed measurements at different concentrations of ATP, ranging from 0 to 5000 µm (past saturation) (**Figure** **3**A). We simulated the phenomena observed on the fiber (Figure S1, Supporting Information) and formulated a mathematical model wherein the population of microtubules on the tip of the fiber decays exponentially but is moderated by a second term, the inverse exponential of microtubules re‐joining the fiber core – Equation (1).

(1) $$I=I_{0}\;e^{-\Lambda t}+Z\left(I_{0}-I_{0}e^{-\Lambda t}\right)$$

(2) $$I=I_{0}\;\left(1-Z\right)\;e^{-\Lambda t}+ZI_{0}$$

(3) $$I_{rel}=I/\;I_{0}=\left(1-Z\right)\;e^{-\Lambda t}+Z$$

This second term contains the same decay constant as the first, as they are both related to the same total population, and is modulated by a “re‐joining” factor Z, here modelled as a fitted constant. Z in fact embodies two different phenomena. The first being that microtubules gliding from the core of the fiber tip onto the cladding of the fiber tip, which is also coated with kinesin, no longer contribute to the emission signal. However, they can turn back, rediscover the core, and reinforce the emission signal. We observed no clear evidence of microtubules moving from the tip cladding to the side cladding of the fiber or vice versa; all microtubules were observed to detach completely at the edge of the fiber. The second phenomenon is that microtubules that have completely left the fiber are still then diffusing in suspension around the fiber. We observed that these microtubules can re‐land on the fiber should their random walk happen to bring them back into contact with it. By fitting this model to our APD data, we produce a value for each curve's decay constant, **Λ**. We then equivocate **Λ** to the gliding speed by means of Equation (4) which accounts for the dimensions of the fiber and therein the average distance travelled by a microtubule.

(4) $$V=\frac{\Lambda \;r}{H}$$

here, *r* is the radius of the fiber and *H* is a calibration constant. By plotting our **Λ** values against microtubule gliding speed *V*, which we measured independently using FIESTA tracking software on videos of the fiber tips, we confirm the linear **Λ** – v relationship (Figure 3B). Assuming there is no bundling, this allows us to deduce gliding speed from the decay curves without a microscope – a new method for gliding speed determination.

**Figure 4:**
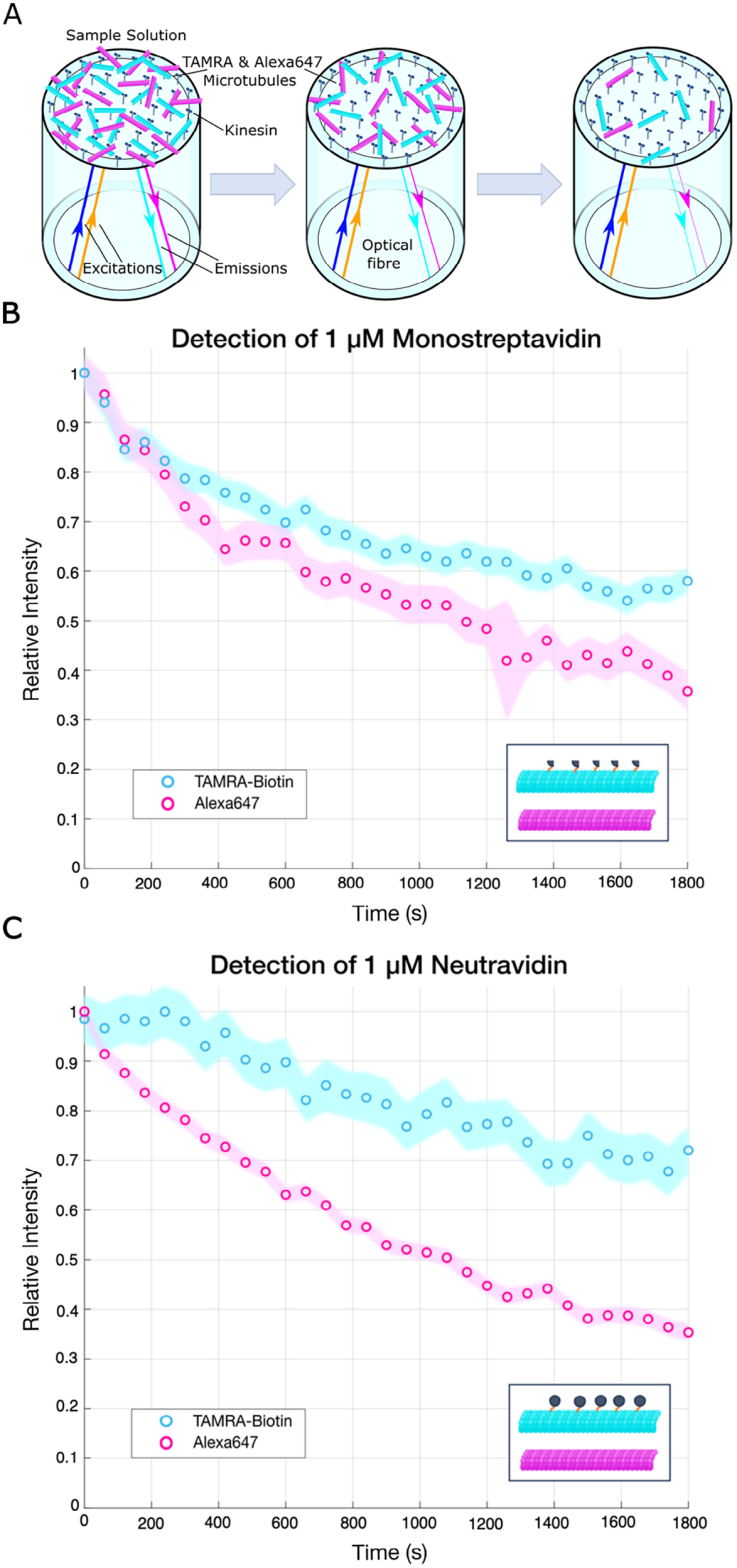
Alexa647 and 40% Biotinylated TAMRA labelled microtubules glide on a fiber tip 1 µm SAvPhire Monomeric Streptavidin solution and 1 µm Neutravidin solution. a) Illustration of TAMRA‐Biotin (cyan microtubules, orange excitation light, cyan emission light) and Alexa647 (magenta microtubules, navy excitation light, magenta emission light) labelled microtubules gliding on the fiber tip and leaving the fiber over time. The filter wheels switch between the excitation and emission filters of each population at every measurement. b) The control Alexa647 labelled microtubule signal decays as expected whereas the biotinylated microtubules with bound Monomeric Streptavidin (MW = 15.5 kDa) are slowed down. Each point is again made up of 198 taken data points with the shutter opening 22 times in 58 s for each set of fluorophore filters, alternating between the two with each measurement. Plotting the 95% confidence intervals shows that the signals are statistically different after 5 min with a two‐sample Kolmogorov–Smirnov test giving *p* < 0.001. c) When Neutravidin (MW = 60 kDa) is bound, the microtubules slow down and also bundle, producing an erratic, very slowly decaying signal. Plotting the 95% confidence intervals shows that the signals are statistically different after 1 min with a two‐sample Kolmogorov–Smirnov test giving *p* < 0.000 001.

In order to utilize the system as a detector, two fluorescently labelled populations of microtubules need to be measured on the fiber simultaneously where one functions as a control (**Figure** **4**A). In order to achieve this, the filter wheels alternate between the TAMRA and Alexa647 excitation/emission filters with each opening of the shutter, opening every 2.6 s for each fluorophore. We tested and subsequently excluded bleaching of both dyes as a factor at our illumination intensity and time scale (Figure S2, Supporting Information). The pure slowdown mode of detection was demonstrated using Alexa647 labelled microtubules as the control as well as biotinylated–TAMRA microtubules and 1 µm SAvPhire Monomeric Streptavidin (MW = 15.5 kDa) (Figure 4B). Due to its small size, this analyte causes a very slight decrease in speed even at high concentrations (1 µm), measured as (98 ± 51) nm s^−1^ in a glass coverslip flow cell (Figure S3, Supporting Information). The difference in speed causes the population numbers, and therein fluorescence signals, to diverge, becoming statistically differentiable after 5 min. Applying our mathematical model gave a difference in gliding speed of (88 ± 77) nm s^−1^ on the fiber, corroborating the difference seen in the flow cell. The bundling mode of detection was demonstrated using biotinylated microtubules and 1 µm Neutravidin (MW = 60 kDa) (Figure 4C; Movie S2, Supporting Information). This analyte initiates a much greater slowdown due to its larger size, measured as (462 ± 62) nm s^−1^ in a flow cell (Figure S3, Supporting Information), as well as causing microtubules to bundle through its four binding sites. This bundling prevents microtubules from leaving the fiber; holding them on the tip where they continue to contribute to the fluorescence signal, which no longer decays in agreement with the mathematical model. As can be seen, the curves become statistically differentiable after 1 min.

**Figure 5:**
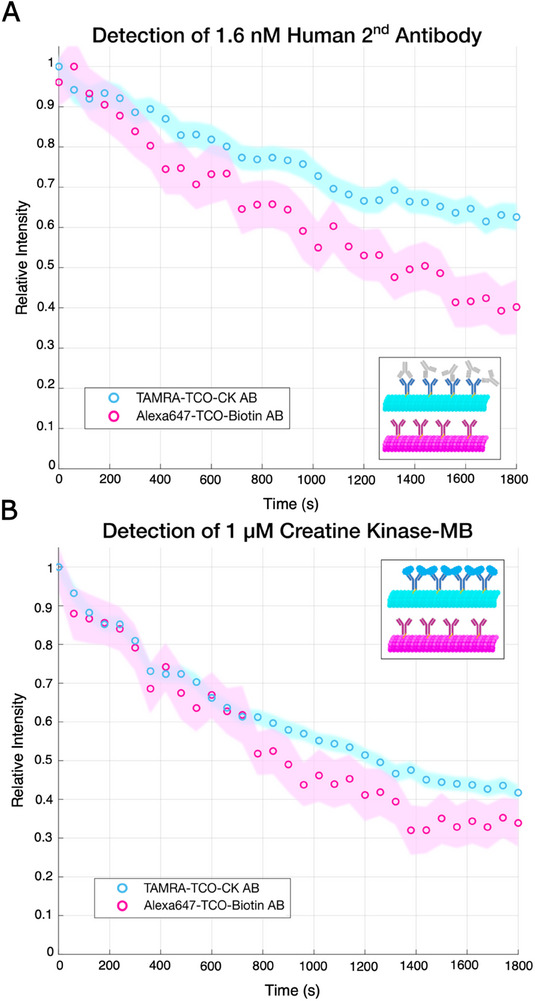
Relative intensity signal of 50% Alexa647 50% TCO tubulin microtubules functionalized with Goat anti‐Biotin antibody and 50% TAMRA 50% TCO tubulin microtubules functionalized with Human anti‐Creatine Kinase‐MB antibody with 1.6 nm Human 2 nd antibody and 1 µm Human Creatine Kinase‐MB. a) The secondary antibody causes a secondary slowdown in the TAMRA population as well as causing minor bundling. Plotting the 95% confidence intervals shows that the signals are statistically different after 6 min with a two‐sample Kolmogorov–Smirnov test giving *p* < 0.01. b) Creatine Kinase‐MB causes a less significant slowdown and there is no bundling factor. Plotting the 95% confidence intervals shows that the signals are statistically different after 15 min with a two‐sample Kolmogorov–Smirnov test giving *p* < 0.03.

To detect an unlabeled analyte, we functionalized microtubules with the corresponding antibodies by tetrazine‐TCO conjugation, an example of inverse‐electron‐demand Diels–Alder addition.^[^ ^10^, ^26^ ^]^ The antibody conjugation is, in itself, cargo loading of microtubules and, therefore causes a slowdown through the roadblock effect. This means that the control population must be conjugated with a different antibody, unrelated to the sample in which the detection will occur or corresponding to a target too small to further decrease gliding speed. Here we use Goat anti‐Biotin. The addition of the target analyte then causes a second subsidiary slowdown affecting only the measurement population, measured in a flow cell as (181 ± 47) nm s^−1^ at 1.6 nm secondary antibody concentration (Figure S4, Supporting Information). Primary antibody binding to the microtubules was confirmed by measuring population slowdown as well as with fluorescent secondary antibodies on gliding assays in flow cells (Figure S5, Supporting Information). Nonfluorescent polyclonal secondary antibodies were detected on fiber at a concentration of 1.6 nm (**Figure** **5**A). This detection demonstrates the efficacy of the primary antibody functionalized system. The signals are statistically differentiable after to 6 min. Finally, we detect the heart attack marker Creatine Kinase‐MB (MW = 82 kDa) as evidence of potential use beyond a strictly academic setting (Figure 5B). We demonstrate proof of concept with detection at 1 µm concentration, which generated a slowdown of (103 ± 54) nm s^−1^ in the flow cell (Figure S6, Supporting Information). On fiber the signals are statistically differentiable after 15 min, a realistic response time for a test administered at the point of care. This detection of an unlabelled medical analyte in solution demonstrates the efficacy of the detector and paves the way for improving upon the detection limit to allow for detection in real samples, i.e., blood, as well as tweaking the design to be able to screen for multiple analytes simultaneously.

## 3 Discussion

Here, we demonstrate a detector capable of ≈1 nm range detection. We suggest that this detector would be best put to use at point‐of‐care given its small requisite sample size and real‐time feedback with detection possible within 15 min. The system is significantly cheaper and smaller than fluorescence microscopy setups, with the prototype's components summing to less than €10 000. In order for gliding assays to be effective in blood, the sample must be diluted a 100fold;^[^ ^27^ ^]^ this currently puts some analytes out of reach, however, it allows for a small sample size – a pinprick of blood will do – which suits point‐of‐care devices better. Detection limits in a roadblock gliding assay can potentially be improved in a number of ways; our first suggestion would be to substitute our primary antibodies for Fab fragments. This would cause a smaller initial slowdown in the control and measurement populations due to their smaller size, 50 kDa compared with 150 kDa antibodies, and thereby increase the magnitude of the secondary slowdown due to the improved ratio of molecular weight increase with analyte binding. Also, this would cause the analyte to be closer to the microtubule, further hindering kinesin binding and increasing slowdown. Second, our TCO‐tetrazine conjugation currently has 9 PEG linkers; this number could be decreased, bringing the primary antibody or Fab even closer to the microtubule; One of the contributing factors to the large slowdown with Neutravidin is the short EZ‐Link NHSester‐Biotin linker used. This would, we believe, bring the detection limit into the pm range. The use of aptamers instead of antibodies as the analyte binding mechanism should also be investigated. Finally, the fiber could be removed from the analyte solution after a set time and placed into a second detection antibody containing a motility solution. This would create sandwich assays on the gliding microtubules, which would greatly increase the roadblock size and initiate bundling, thus amplifying the signal change.

It is interesting to note that the size of the roadblock effect and, therefore, the detection limit is highly dependent on the bioconjugation method, i.e., linker length, as well as the analyte in question. Secondary antibodies are detected at 1.6 nm, with a theoretical detection limit in the pm range as shown in the flow cell and supported in literature, where the bundling method is also used to detect microvesicles for the diagnosis of leukemia.^[^ ^11^ ^]^ This would potentially allow the detector to be used for monitoring the maintenance of therapeutic antibody through concentrations in treatments for autoimmune diseases such as Crohn's where levels of the antibody Infliximab need to be kept above 3–70 nm in blood; a fiber‐optic/antibody sandwich assay‐based biosensor with a superior detection limit of 15 pm but a longer assay time of ≈35 min being used to this effect.^[^ ^25^ ^]^ Creatine Kinase‐MB, however, did not produce a >100 nm s^−1^ slowdown below µm concentration, far above the detection limit of commercially available point‐of‐care CK‐MB devices such as the Siemens healthcare Stratus CS Acute Care System with a detection limit of 3.6 pm.^[^ ^28^ ^]^ However, our device is competitive with regard to time and specificity − <15 min for our device versus 14 min for Siemens which can also detect up to four heart attack markers in 26 min – as well as being a considerable improvement on cost (<€10 000) versus $51337.99. We believe that our high detection limit of Creatine Kinase is due to a number of factors – first the roadblock effect is dependent not only on analyte size but also on shape and charge.^[^ ^14^ ^]^ Potentially then, Creatine Kinase‐MB, which is negatively charged at physiological pH, has a lesser impact than more neutral secondary antibodies as it is electrostatically repelled from the negatively charged microtubule surface and so hinders kinesin less. Another point is the proximity of the binding site of the second molecule relative to the microtubule, with polyclonal secondary antibodies potentially binding closer and creating a more formidable roadblock. Finally, the dissociation constant of the analyte will affect the quantity bound, as will the ratio of fluorescently labelled tubulin to TCO.

A major advantage to the method described is its potential in future work for multiplexing and wide‐ranging analyte detection as multiple fluorescent, antibody‐functionalized populations of microtubules (beyond the demonstrated two) could readily be utilized on the same fiber tip (with the addition of extra emission filters) and multiple optical fibers in a bundle could be scanning a sample for analytes in parallel. This would also allow the fibers to be used for screening tests. Moreover, fibers, once used can be cleaned by the method suggested for the regeneration of molecular motor‐based nanodevices;^[^ ^29^ ^]^ and can also be repaired if cracked by polishing. Provided that they were cleaned correctly, we observed only little difference in performance across eight different fibers used in our experiments (Figure S7, Supporting Information). With further work done to improve the detection limit, we believe our detector will be a low‐cost solution for in situ, rapid, wide‐ranging, analyte detection and screening.

## 4 Experimental Section

### Optic Fiber

Silica/PolyClas Optical fiber JTFLH200‐Low‐OH multimode optical fibers with SMA905 connectors (Laser Components, Germany). The optical fibers utilized in this study were terminated to a length of 2 m. One end was polished and left bare, while the other end was secured to an SMA connector through a termination process and polished. Fibers were photobleached overnight, saturating fluorophores in the fiber in order to minimize and stabilize the background autofluorescence signal. Prior to experimentation, the fibers were thoroughly cleaned by sonicating in subsequent solutions of activated proteinase K (200 µg mL^−1^) for 1 h to cleave surface adsorbed proteins, 5 mm PMSF for 5 min, 5%SDS for 5 min, 20% Mucasol for 15 min and Ethanol for 10 min.^[^ ^28^ ^]^

### Microtubule and Kinesin Preparation

Microtubules were polymerized from 4 mg mL−^1^ porcine brain tubulin,^[^ ^30^ ^]^ – labeled with TAMRA or Alexa647, or functionalized with NHSester‐biotin or NHSester‐PEG_4_‐TCO – in BRB80 with 5 mm MgCl_2_, 1 mm GTP, and 5% dimethyl sulfoxide (DMSO) at 37 °C for 30 min. The microtubules were stabilized and diluted 20‐fold in BRB80 containing 10 µm taxol. Full‐length Drosophila melanogaster kinesin‐1 motor proteins were expressed in insect cells and purified as previously described.^[^ ^31^ ^]^

### Antibodies and Analytes

Goat Anti‐Biotin Antibody (Sigma–Aldrich, Germany) and Human Anti‐Creatine Kinase‐MB antibody produced in *E.Coli* (Scripps, California, USA) were conjugated to microtubules via 6‐Methyl‐Tetrazine‐PEG5‐NHSester (Jena Bioscience, Jena, Germany). Anti‐Sheep FITC secondary antibody produced in rabbit (Thermofisher Scientific – Pierce, USA) and Anti‐Human Alexa Fluor 488 secondary antibody produced in goat (Thermofisher Scientific, USA) were used to verify the primary antibody – microtubule conjugation and unlabelled anti‐Human secondary antibody (Invitrogen, Germany) was used as an analyte. Further reagents used: i) Neutravidin (ThermoFisher, Germany), ii) SAvPhire Monomeric Streptavidin (Sigma–Aldrich, Germany), iii) EZ Link NHSester‐biotin (ThermoFisher, Germany), iv) Human Creatine Kinase‐MB (Scripps, California, USA) Antibodies were conjugated to microtubules in the manner previously elucidated;^[^ ^10^ ^]^ with TCO‐functionalized‐tubulin microtubules.^[^ ^32^ ^]^ 5 µL of Anti‐Biotin Goat Antibody and 5 µL Anti‐Human‐CKMB *E.coli* Antibody in PBS (5 mg mL^−1^, ≈33 µm) were incubated with 0.5 µL of 6.5 mm 6‐Methyl‐Tetrazine‐PEG_5_‐NHSester in DMSO each overnight at 4 °C. The excess tetrazine was removed by the Zeba Biotin & Dye spin removal column (Thermofisher), increasing the volume of each sample to 50 µL with BRB80T and centrifuging at 2000 rcf for 2 min. 5 µL of each antibody was then added to 5 µL of their respective fluorescently labelled microtubules at room temperature for 1 h to ensure saturation.

### Flow Cell Assembly

Flow cells were assembled by placing three parallel strips of parafilm (25 × 2 mm^2^) spaced apart by ≈2 mm on a 22 × 22 mm^2^ coverslip (Corning) and a smaller 18 × 18 mm^2^ coverslip (Corning) on top. Both coverslips were treated with the Easy Clean procedure. This sandwiched setup was assembled on a thin tissue (Kimtech) and placed on a heating block at 80 °C for ≈15 sec to melt the parafilm strips, thereby creating two imaging channels. The coverslip on top was gently pressed at the sites of the parafilm strips to release any bubbles and ensure leakproof channels.

### Tip Cell Assembly

The tip cell consists of a pipette tip sheared to slightly larger than the circumference of the fiber and glued to a PEGylated coverslip using UV‐activated adhesive (Norland 61). Solutions were delivered into the TipCell via a syringe.

### Gliding Assay Mounted on Optical Fibre (GLAMOR) Protocol

An optical fiber was dipped into successive solutions in Brinkley Reassembly Buffer 80 mm (BRB80; adjusted to pH 6.9 with KOH) composed of 80 mm 1,4‐piperazinediethanesulfonic acid (PIPES), 1 mm EGTA, and 1 mm MgCl_2_. BRB80. The first contained casein (0.5 mg mL^−1^) which was allowed to adsorb to the surface for 5 min. The fiber was then dipped into Kinesin solution (4 µg mL^−1^ kinesin‐1, 0.2 mg mL^−1^ casein, 10 mm dithiothreitol and 10 µm ATP) and allowed to adsorb for 10 min. To measure the background the fiber was placed into a low ATP motility solution (20 mm d‐glucose, 55 µg mL^−1^ glucose oxidase, 11 µg mL^−1^ catalase, 10 mm dithiothreitol, 10 µm taxol and 10 µm ATP), and the background signal was measured for 2 min. The fiber was then removed and dipped into microtubule solution (20 mm d‐glucose, 55 µg mL^−1^ glucose oxidase, 11 µg mL^−1^ catalase, 10 mm dithiothreitol, 10 µm taxol, 10 µm ATP and stabilized microtubules (2 µm polymerized tubulin)) for 10 min. Finally, unbound microtubules were washed away by dipping again into Low‐ATP Motility Solution before placing the fiber into the analyte solution ((20 mm d‐glucose, 55 µg mL^−1^ glucose oxidase, 11 µg mL^−1^ catalase, 10 mm dithiothreitol, 10 µm taxol, 5 mm ATP (or alternative ATP concentration) and the analyte at the chosen concentration).

### Gliding Assays in Flow Cells

A 0.5 mg mL^−1^ solution of casein in BRB80 was flowed in and incubated for 2 min. Kinesin‐1 solution (as above) was then added and incubated for 10 min. Finally, a motility solution (1 mm ATP, 20 mm D‐glucose, 20 mm glucose oxidase, 10 mm catalase, 10 mm DTT, and 10 µm taxol in BRB80) with the microtubules was added. Excess unbound microtubules were removed by flushing the motility solution without the microtubules and containing any relevant analyte in the desired concentration.

### Detector Setup

The setup consisted of: i) Light Source: SOLA Light Engine (Lumencor, USA), ii) Collimator: (Thorlabs, Germany), iii) Filter Wheels: (Visitron Systems, Germany), iv) Filters, Filter Cubes and Dichroic Mirror: (Thorlabs, Germany), v) Excitation filters – TAMRA 542/20 nm, Alexa647 642/40 nm, vi) Emission Filters – TAMRA 578/22 nm, Alexa647 700/75 nm, vii) Dichroic Mirror – Reflect at 500–533 & 617–652 nm, Transmit at 564 – 591 & 665 – 725 nm, viii) Fiber: Silica/PolyClas Optical fibre JTFLH200‐Low‐OH multimode optical fibers with SMA905 connectors (Laser Components, Germany), ix) Avalanche Photo Diode: COUNTT100 (LaserComponents, Germany), x) Software: LabView

### Imaging

Image acquisition was performed using an inverted fluorescence microscope Axio Observer Z1 and a 40 × oil α Plan‐Apochromat NA = 1.3 objective (Zeiss, Germany). The data was recorded with an electron multiplying charge‐coupled device (EMCCD) camera (iXon Ultra EMCCD, DU‐897U, Andor) having a pixel size of 16 µm. Images were acquired with an exposure time of 100 ms using MetaMorph (Molecular Devices, USA).

### Data Analysis

To analyze videos of gliding microtubules on fiber in‐house tracking software FIESTA, which was built in MATLAB was used. For determining the speed of microtubules in flow cells the AutoTipTrack program was used.^[^ ^33^ ^]^ For calculating the intensity of the microtubules on the fiber to corroborate the avalanche photodiode signal ImageJ was used. The avalanche photodiode signal was measured every ten milliseconds and processed using a program written in LabView (Figure S8, Supporting Information).

### Simulation Model

A Monte–Carlo simulation approach, developed from previous work^[^ ^34^, ^35^, ^36^, ^37^ ^]^ and implemented using MATLAB was employed (Mathworks, Natick, MA). The source code was publicly available under a permissive BSD 3‐clause license on github.^[^^]^ Briefly, only the microtubule tip was simulated because the tip finds the next motor and moves along that path. The path of the microtubule was generated using normally distributed angular updates in gliding direction *∆θ* depending on the persistence length *(L_p_)* and distance between time steps.

(5) $$\mathrm{Angular}\;\mathrm{change}\;\mathrm{per}\;\mathrm{step}:\Delta \theta =\sqrt{\frac{v_{f}*\Delta t}{L_{p}}}$$

here, *v_f_* is the sliding velocity, *Δt* is the time interval (10 ms) between updates in the gliding direction, and *L_p_* is the microtubule persistence length. To simulate the gliding motion of microtubules, changes in direction were randomly generated from a normal distribution. If the microtubules move beyond the defined boundaries of the motility‐supporting zone, they were detached with a specified probability of 0.8. Otherwise, they were directed along the boundary and continue gliding according to the algorithm specified above. This means that they have a certain probability to move toward the centre or leave again. Should a microtubule leave it then diffuses in a large zone “above” and encompassing the motility‐supported zone with a specified probability to land of 0.01 per second should it still be “above” the motility‐supported zone.

The simulation code: a) creates a guiding structure from the design file of the fiber tip and a large surrounding area b) simulates the microtubules by giving them properties such as speed, persistence length, leaving probability, and landing probability.

## Conflict of Interest

The authors declare no conflict of interest.

## Supporting information



Supporting Information

Supplemental Movie 1

Supplemental Movie 2

## Data Availability

The data that support the findings of this study are available from the corresponding author upon reasonable request.
