# Supplementary material for: Microscope‐Free Analyte Detection Based on Fiber‐Optic Gliding Motility Assays
Source: Small. 2025 Apr 16;21(22):2411836. doi: 10.1002/smll.202411836 (PMC12138846; doi:10.1002/smll.202411836)
Supplement: Supplementary file 1 — Supporting Information [file SMLL-21-2411836-s002.pdf]

NANO · MICRO
small

Supporting Information

for *Small*, DOI 10.1002/smll.202411836

Microscope-Free Analyte Detection Based on Fiber-Optic Gliding Motility Assays

*Henry Carey-Morgan, Nabarun Polley, Till Korten, Claudia Pacholski and Stefan Diez**

Supplementary Information

Microscope-free analyte detection based on fibre-optic gliding motility assays

Henry Carey-Morgan[1], Nabarun Polley[2], Till Korten[1,5], Claudia Pacholski[2], Stefan Diez[1,3,4]

[1] *B CUBE – Center for Molecular Bioengineering, TUD University of Technology Dresden, 01307 Dresden, Germany*

[2] *University of Potsdam, Institute of Chemistry, Physical Chemistry – innoFSPEC, Am Mühlenberg 3, 14476 Potsdam, Germany*

[3] *Max Planck Institute for Molecular Cell Biology and Genetics, 01307 Dresden, Germany*

[4] *Cluster of Excellence Physics of Life, TUD University of Technology Dresden, 01062 Dresden, Germany*

[5] *current addresse: Helmholz Zentrum Dresden Rossendorf HZDR, Dresden*

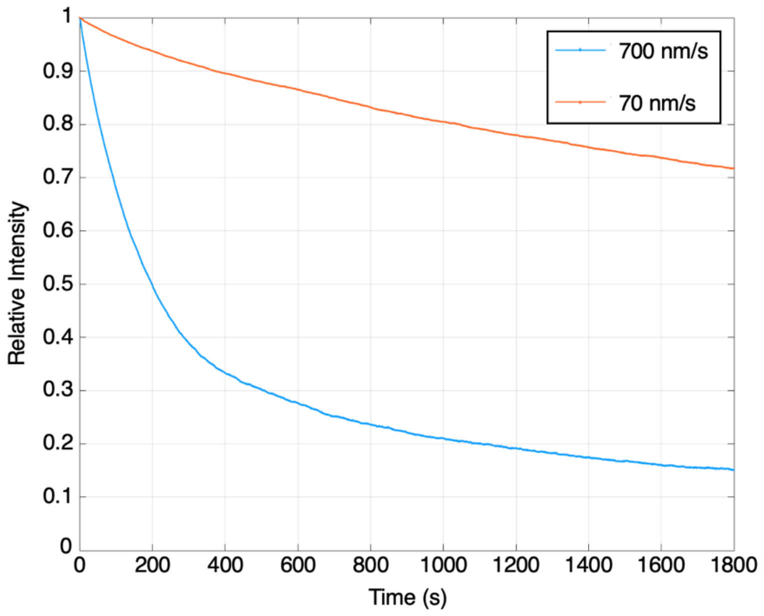

Figure S1. Simulations of microtubules on the tip of a fibre moving at different speeds with a possibility to re-join the fibre core. We simulated microtubules gliding on the tip of a 200 μm optical fibre and their numbers decreasing over time as they reached the edge. Microtubules re-joining the core and thereby contributing once again to the emission signal were observed in experiments through two mechanisms, first microtubules that glided off of the core of the fibre onto the cladding could change direction and return to the core; second, microtubules diffusing in solution could re-join should their random walk bring them back into contact with it. In the simulations we included the possibility to glide back on to the core by having some microtubules 'bounce' off of the edge of the fibre on contact – a probability specified by the 'WallDetach' parameter. Re-joining the fibre through diffusion was included by simulating microtubules' random walk displacement in solution with respect to the fibre and including a probability – the Rebirth parameter – that they successfully re-joined should they still be 'above' the fibre and not diffused away (**Materials and Methods**). The signals simulated at different speeds allowed us to anticipate the magnitude of speed decrease required to differentiate signals and correspond approximately with measured values.

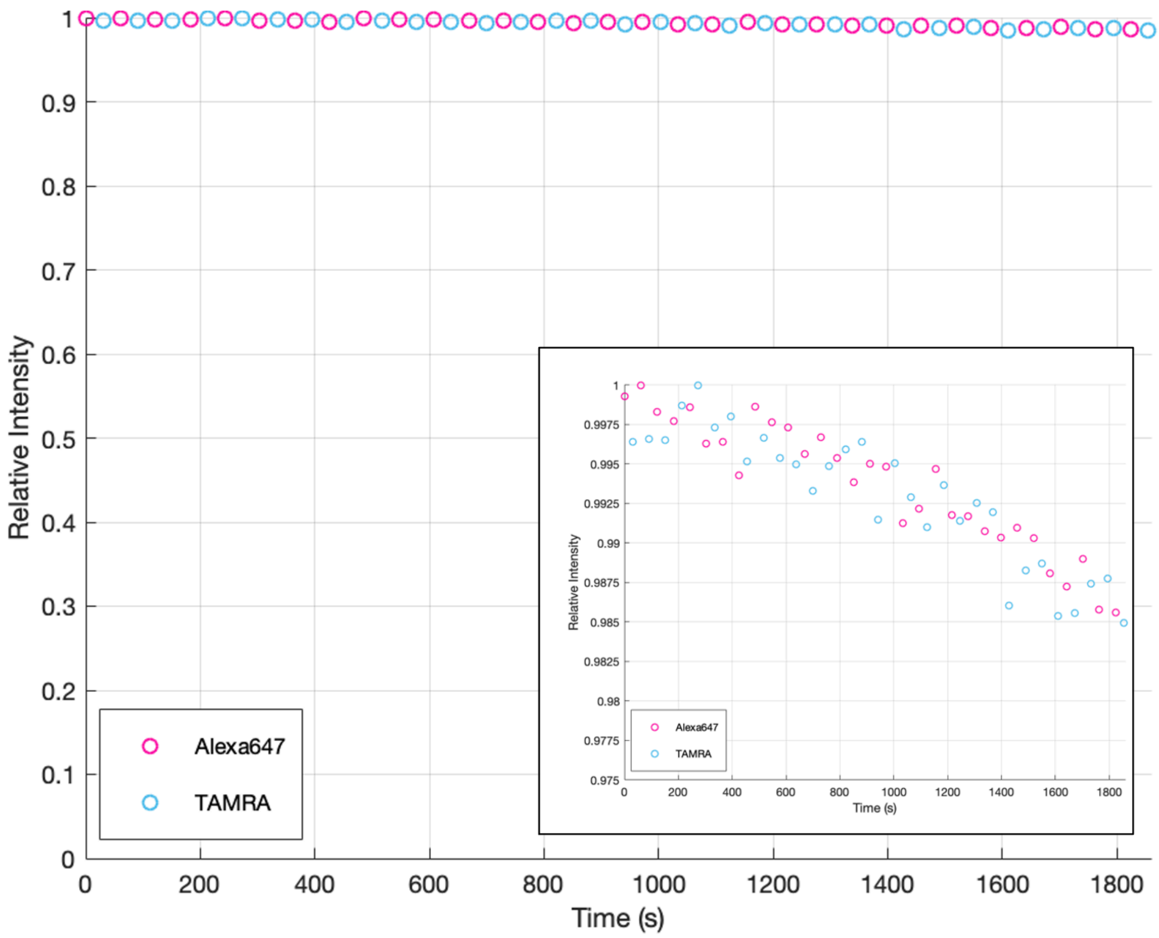

Figure S2. APD signal of Alexa647 and TAMRA labelled microtubules in solution without ATP each showing a 1.5% decrease in signal after half an hour. As photobleaching is minimal and equivalent in both fluorescent populations it is excluded in the treatment of population decay curves. Inset shows same graph zoomed in on y axis.

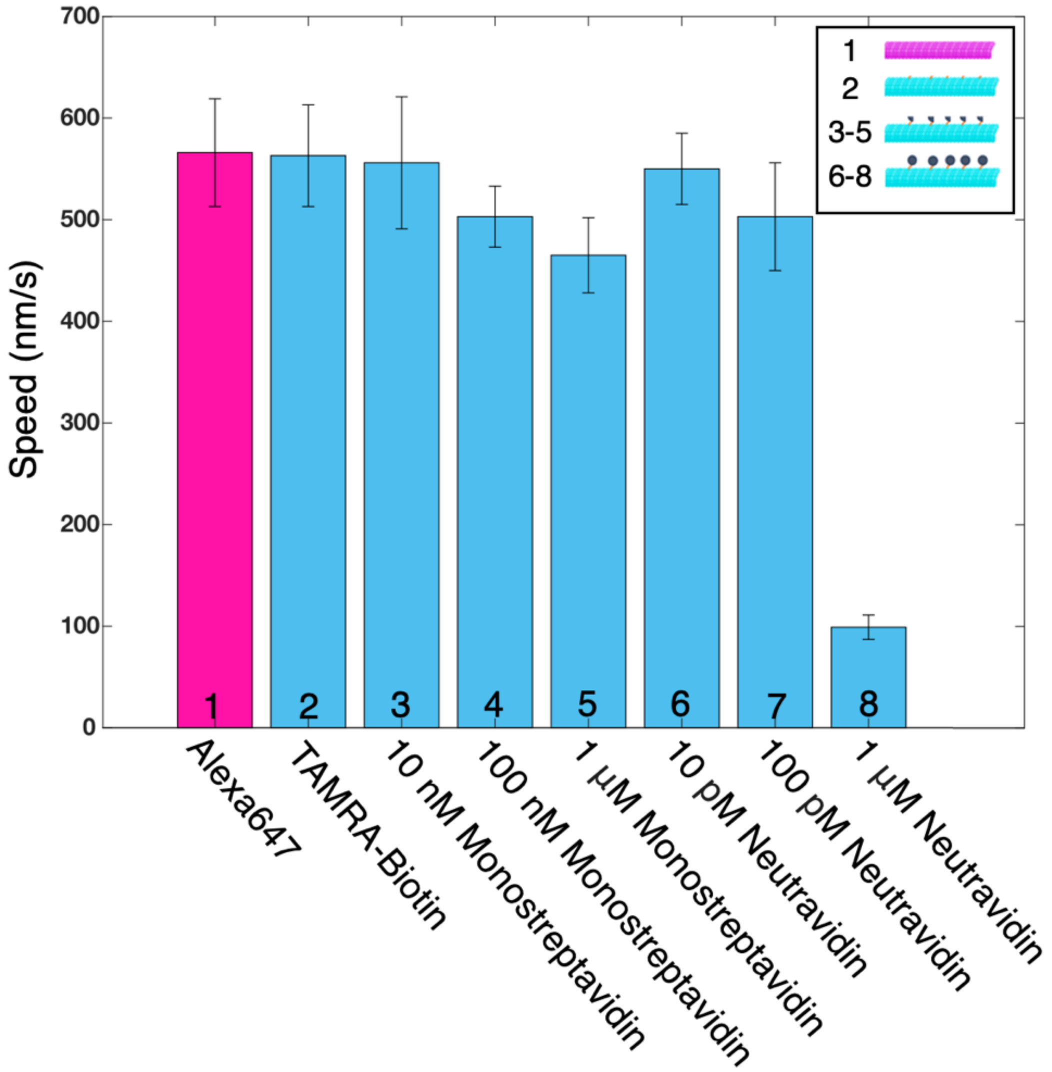

Figure S3. The roadblock effect demonstrated in flow cells with 40% biotinylated microtubules. Whilst biotin itself is too small to function as a roadblock (2), biotinylated microtubules will slowdown in the presence of SAvPhire™ Monomeric Streptavidin (3-5) – with 1 μM concentration causing a slowdown of (98 ± 51) nm/s (5) – and Neutravidin, which causes a larger slowdown of (462 ± 62) nm/s at a concentration of 1 μM (8). Due to its larger size, Neutravidin also possesses a lower detection limit, causing a slight decrease in speed as well as bundling at 100 pM (7), whereas Monostreptavidin showed no sign of hindering gliding below 100 nM (4)

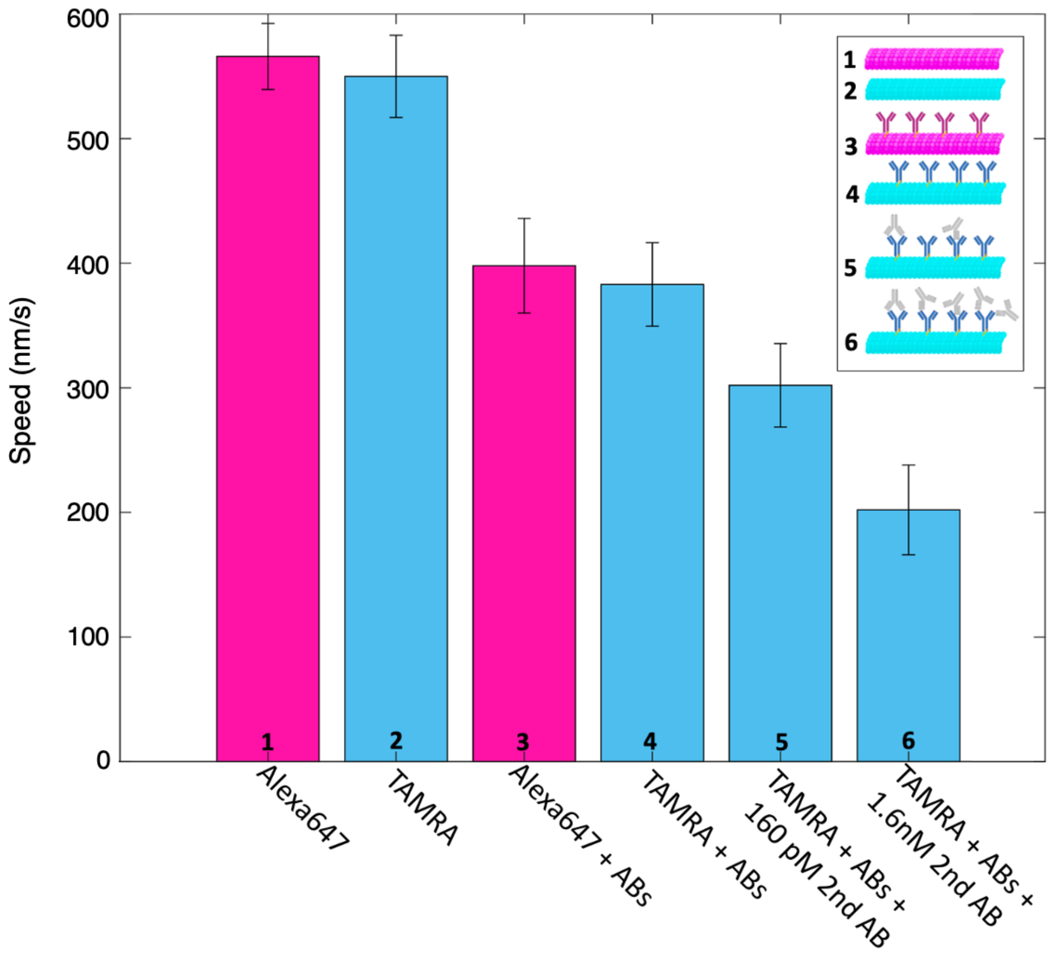

Figure S4. Detection of secondary antibodies with antibody functionalised microtubules in flow cells through decrease in gliding speed. Alexa647 and TAMRA labelled microtubules with 50% TCO tubulin glide at the same rate (1 & 2). These populations, functionalised with Goat anti-Biotin and Human anti-Creatine Kinase-MB in the same concentration by tetrazine-TCO binding, slow down by the same amount – this allows one to function as a control (3 & 4). Addition of secondary antibodies, or a target analyte, causes an additional secondary slowdown which is the phenomena underpinning a detection; greater concentration causes a greater slowdown, shown here with unlabelled polyclonal secondary antibodies decreasing speed by (81 ± 49) nm/s and (181 ± 47) nm/s at 160 pM and 1.6 nM concentrations, respectively (5 & 6).

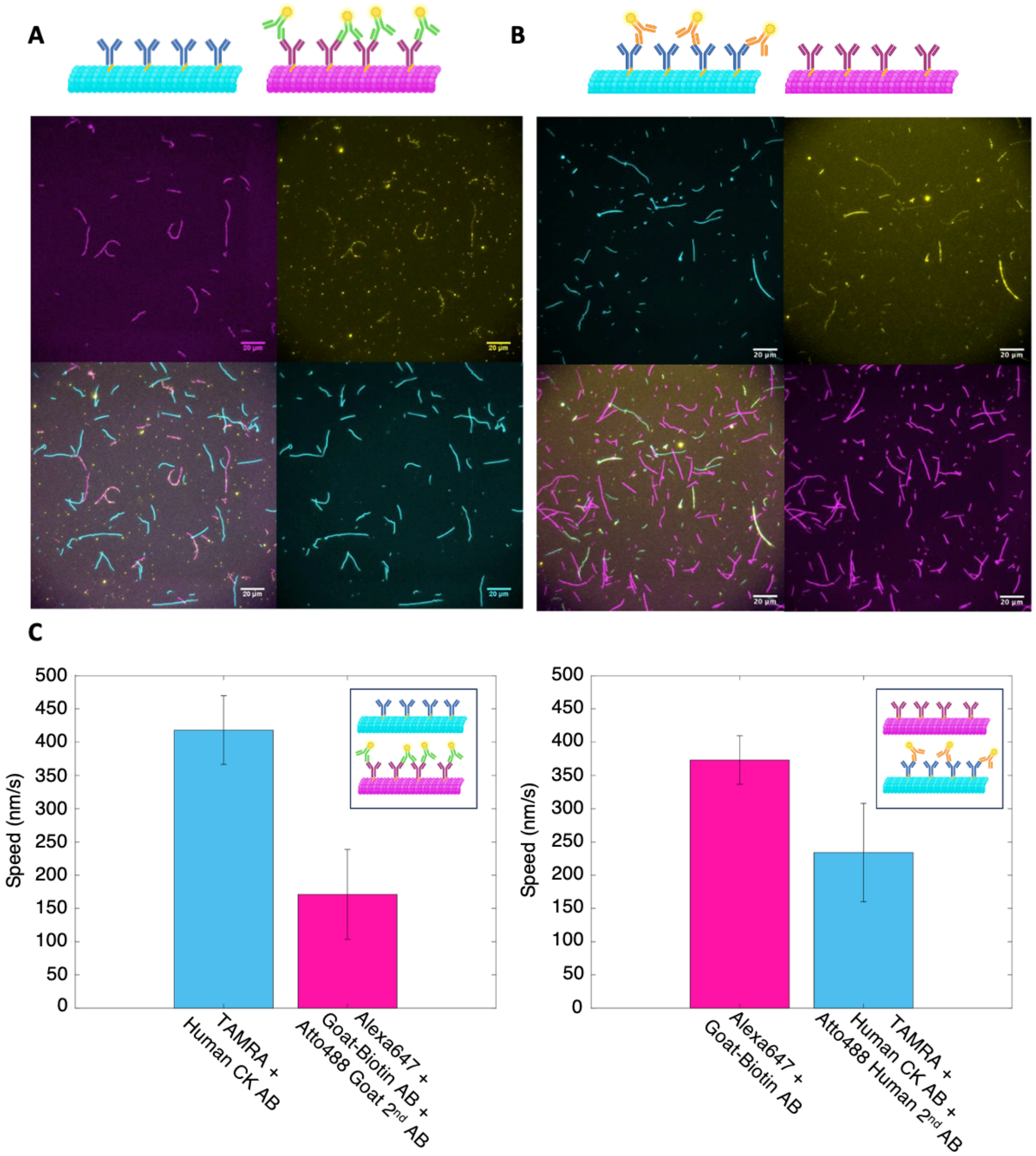

Figure S5. Fluorescent secondary antibody binding to antibody functionalised microtubules. A) Moving clockwise from top left, Alexa647-Goat anti-Biotin labelled microtubules (magenta) are coated in Goat secondary antibody (yellow) whilst TAMRA-Human CK-MB microtubules (cyan) are unmarked by secondary antibody, final panel is merged view. Scale bar 20 μm**. B**) Moving clockwise from top left, TAMRA Human Anti-CK-MB labelled microtubules (cyan) are coated in human secondary antibody (yellow) whilst Alexa647-Goat anti-Biotin labelled microtubules are unmarked, final panel is merged view. Scale bar 20 μm. **C**) Microtubules with secondary antibodies experience a secondary slowdown as shown in both populations.

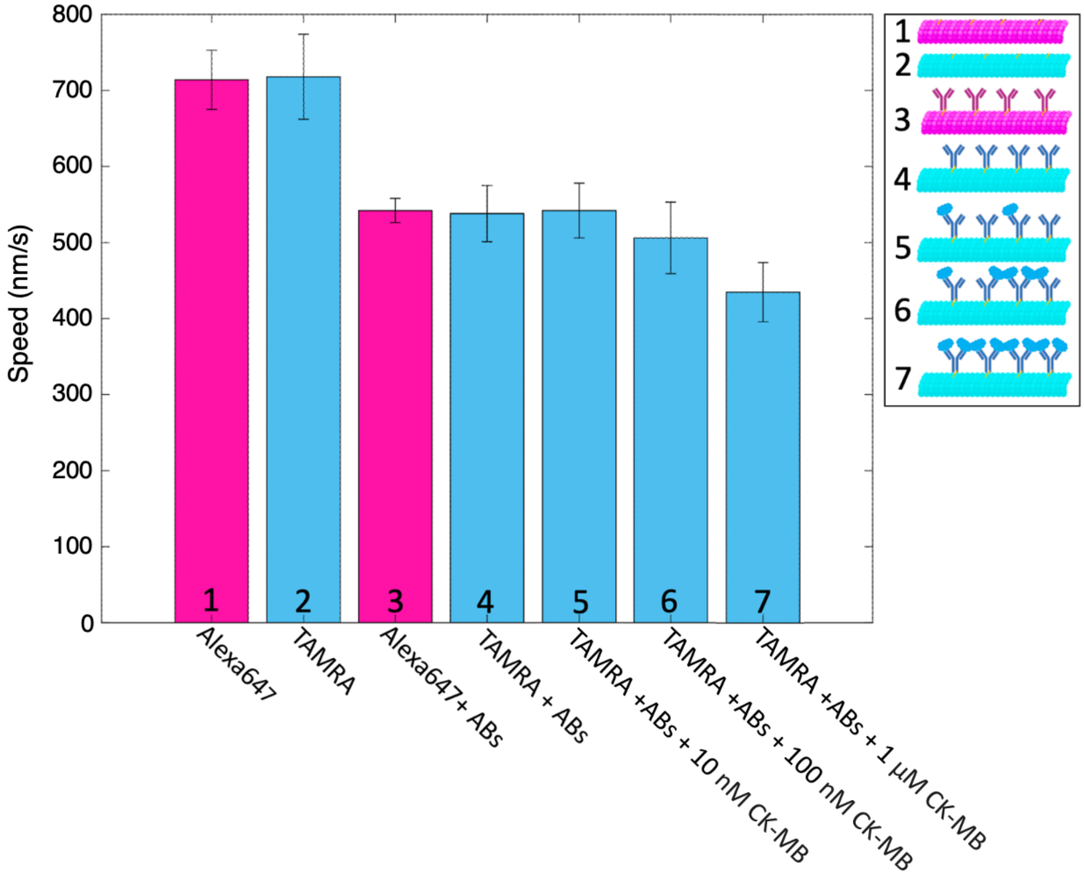

Figure S6. Detection of Creatine Kinase-MB in flow cells through decrease in gliding speed. Alexa647 and TAMRA labelled microtubules with 50% TCO tubulin glide at the same rate (1 & 2). These populations functionalised with Goat anti-Biotin and Human anti-Creatine Kinase-MB antibody respectively in the same concentration by tetrazine-TCO binding will slowdown by the same amount – this allows one to function as a control (3 & 4). Addition of Creatine Kinase-MB causes an additional secondary slowdown; greater concentration causes a greater slowdown, decreasing speed by (32 ± 60) nm/s and (103 ± 54) nm/s at 100 nM and 1 µM concentrations, respectively (6 & 7).

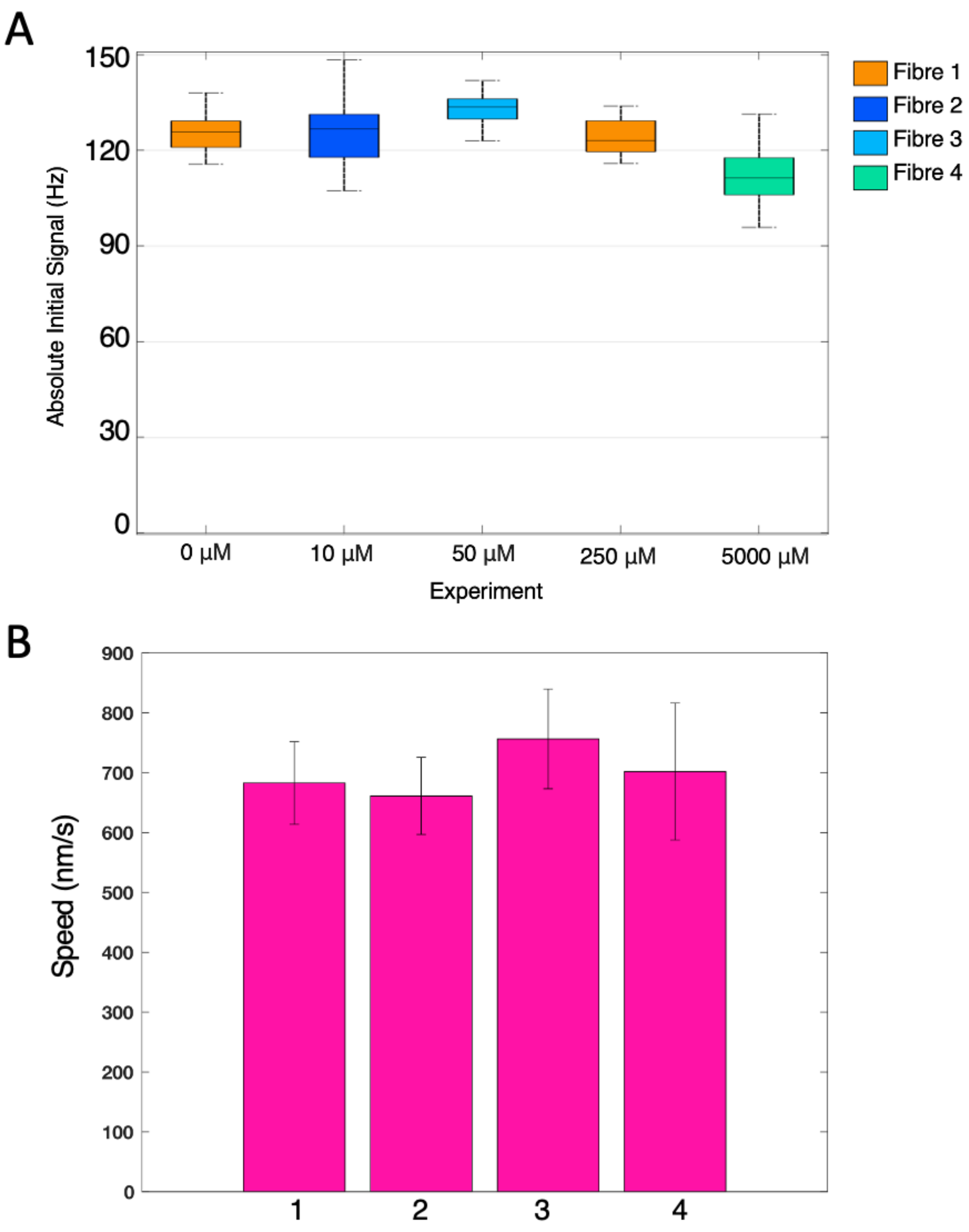

Figure S7: Comparison of assays across 8 different fibres. A) The absolute initial signal values of the five different ATP concentrations used in **Fig. 3**. As can be seen the initial signals are similar albeit with small difference with some fibres, perhaps due to differing signal attenuation caused by damage along the fibre length or different microtubule density loading onto the fibre. Fibre1, which was cleaned and re-used, gives almost the exact same value. **B)** The speeds measured on four different fibres of the Alexa647 control population in the Monostreptavidin (1 & 2) and Neutravidin (3 & 4) experiments (**Fig. 4)**, using our mathematical model, with a repeat each. As can be seen the control population reliably glides at ~700 nm/s on each fibre.

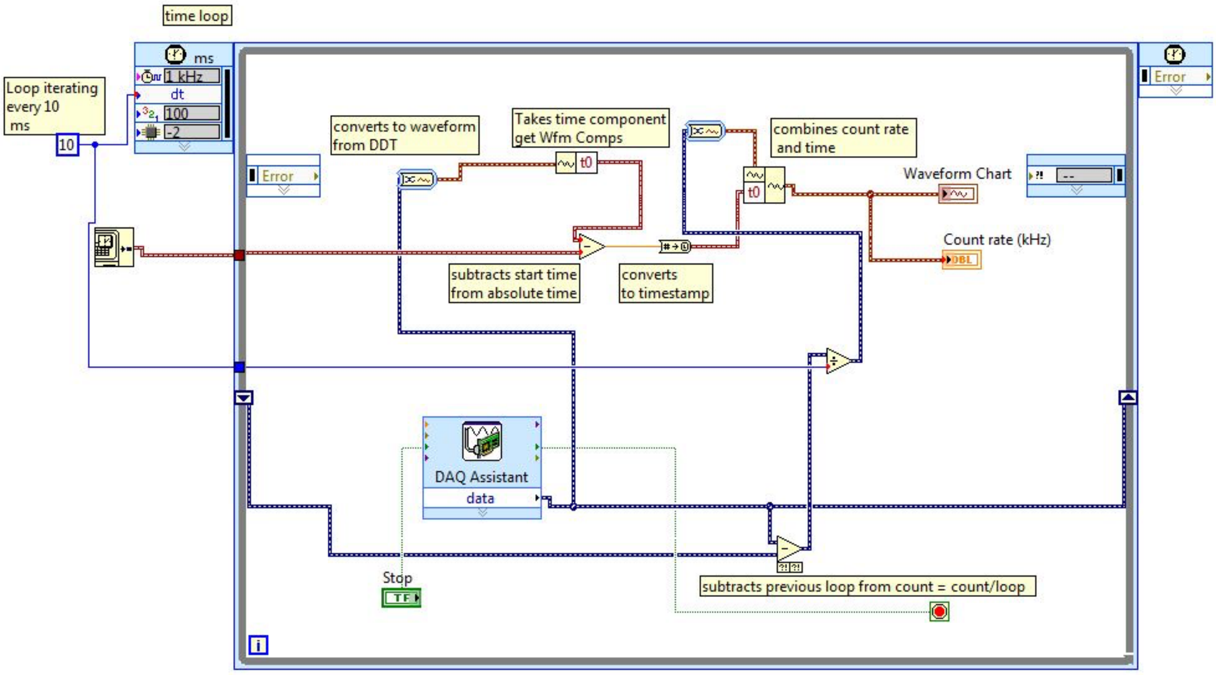

Figure S8: LabVIEW software controlling COUNT®T100 avalanche photodiode. The avalanche photodiode counts photons arriving at the sensor which generate corresponding electrical pulses, registered as 'edges' by the DAQ Assistant. Every 10 ms the number of edges is recorded and converted to an output measurement of Count Rate in kHz. This is shown in a waveform chart that grows with time as well as numerically.
